# Microstructural Modification and Sorption Capacity of Green Coffee Beans

**DOI:** 10.3390/foods13213398

**Published:** 2024-10-25

**Authors:** Weixue Dong, Yutaka Kitamura, Mito Kokawa, Taroh Suzuki, Rasool Khan Amini

**Affiliations:** 1 Graduate School of Science and Technology, University of Tsukuba, Tsukuba 305-8572, Ibaraki, Japan; dongweixue1995@gmail.com; 2 Institute of Life and Environmental Sciences, University of Tsukuba, Tsukuba 305-8572, Ibaraki, Japan; kokawa.mito.ke@u.tsukuba.ac.jp; 3 Saza Coffee Holdings Ltd., Hitachinaka 312-0043, Ibaraki, Japan; suzuki_taroh@saza.coffee (T.S.); rasool.ameen@gmail.com (R.K.A.)

**Keywords:** green coffee beans, modification, freeze-drying, microwave and puffing, flavor compounds, sorption capacity

## Abstract

To enhance the pore structure of green coffee beans (GCB) and detect the sorption capacity and extraction characteristics of flavor compounds before roasting, this study employed several methods: hot air drying (HD), freeze-drying (FD), 3-levels short-time heating with puffing (SH_1_P, SH_2_P, and SH_3_P), and 3-levels microwave with puffing (MW_45_P, MW_60_P, and MW_75_P). These methods were applied to GCBs pre-soaked in water for different times. The effects of these treatments on color change, porosity, microstructure, citric acid sorption capacity, and caffeine and chlorogenic acid extraction yield were investigated. Results indicated that, except for GCBs treated with SH_1_P, SH_2_P, SH_3_P, and MW_75_P, all other modified GCBs showed minimal color change. GCBs treated with MW_60_P exhibited favorable pore structures. MW_60_P treatments significantly improved the extraction yield of caffeine and chlorogenic acid. Furthermore, the increased porosity and improved pore size distribution of GCB after MW_60_P resulted in a significant increase in the sorption of citric acid onto modified GCB. The rate of the sorption reaction followed the pseudo-first-order kinetics. In conclusion, MW_60_P are effective treatments for enhancing the microstructure of GCB, improving sorption capacity, and improving the extraction yield of flavor compounds.

## 1. Introduction

Coffee, which belongs to the *Rubiaceae* family genus *Coffea*, is one of the world’s three major beverages alongside tea and cocoa. Unroasted coffee beans, commonly referred to as green coffee beans (GCB), are a significant economic crop and export product. They are primarily classified into two varieties: *Coffea arabica* (arabica) and *Coffea canephora* (robusta). Arabica, which accounts for 60% of the world’s coffee production, originated in the highland regions of Ethiopia. They are known for their smooth texture and rich complexity and are typically cultivated in temperate high-altitude areas. Robusta, the most common variety of *Coffea canephora*, generally exhibits lower acidity, increased bitterness, and a more woody, less fruity flavor compared to arabica. The majority of robusta coffees are grown in Indonesia and Vietnam [1].

The sensory profile and quality of coffee depend on factors such as the variety, origin, postharvest processing, roasting, and brewing. The postharvest processing refers to the process of obtaining GCB from coffee fruit, and common methods include wet, dry, and pulped natural processes [2]. However, these processing methods fundamentally rely on the yeast, bacteria, and other microorganisms present in coffee fruit to facilitate spontaneous fermentation. Similarly, the emerging method-controlled fermentation is also based on this principle [3,4,5].

Roasting is another effective way to achieve the pleasant specialty coffee aroma and flavor. Roasting is a complex process that involves the transfer of energy (from the surface of the GCB to their interior) and mass (water vapor and volatile compounds from the bean to the environment). Time and temperature, the primary parameters affecting the physical and chemical properties of roasted coffee, significantly influence the flavor, aroma, color, and quality of coffee beans by altering the rates of energy and mass transfer. At specific roasting times and temperatures, the diffusion rates of heat and mass are determined by the structure of the GCB, while the density and porosity of the GCB affect the path length and rate of diffusion. The lower porosity of GCB impedes the rapid and uniform transfer of heat from the surface to interior during the roasting process [6,7]. As a result, problems such as partial charring and over-roasting easily arise during roasting [3]. While considerable research has been dedicated to optimizing roasting parameters and monitoring changes in porosity during roasting to control the flavor profile of roasted coffee beans, few studies have changed the microstructure of GCB before roasting.

The types and concentrations of flavor compounds in roasted coffee beans directly impact the final taste of the coffee. Most current research is concentrated on methods such as brewing techniques, grind size, and brewing temperature to improve the extraction yield of flavor compounds [8,9]. Some researchers have used puffing technology as an alternative to roasting, allowing coffee beans to achieve the same results as roasting through high-temperature puffing. The puffed coffee beans exhibit better porosity and higher extraction yield [10]. However, few studies have directly investigated enhancing the extraction yield of flavor compounds by altering the microstructure of GCB before roasting. Hot air drying and short-time heating and puffing offer advantages of low cost and suitability for large-scale processing. However, excessive temperature or prolonged heating time may lead to the loss of certain heat-sensitive flavor compounds in GCB before roasting. Freeze-drying preserves the integrity of flavor and aroma compounds but is more expensive and time-consuming.

The above-mentioned traditional processing can certainly optimize and uphold the flavor profile of coffee beans, but these flavor components are almost entirely derived from the coffee beans themselves. Currently, numerous studies have immersed GCB in solutions containing flavor compounds like glucose, fructose, and organic acids, aiming to introduce exogenous flavor substances. For example, glucose serves as a primary source of volatile flavor compounds in roasted coffee beans, while citric acid is commonly associated with citrus and acidic flavors. Increasing the citric acid content can enhance the fruity flavor characteristics of coffee beans. Fermentation can increase flavor compound concentration in GCB, but it cannot selectively target a single compound [2,4]. By optimizing the sorption capacity of GCB, it is possible to selectively enhance the flavor profiles, leading to a more desirable coffee experience. However, the GCB structure is compact with only a small number of pores [11,12]. Solely employing a normal immersion method makes it challenging to facilitate the maximal sorption of exogenous flavor substances by GCB within a short duration. Lee and Liu soaked GCB in the solution containing flavor compounds under sonication or high-pressure conditions [13,14]. Although this method allows for the introduction of exogenous flavor substances into GCB, it is costly and difficult to achieve large-scale production. Furthermore, due to the relatively low sorption capacity of GCB, this method also requires longer soaking times. Sorption refers to the process through which a substance (sorbate) becomes attached to a solid phase (sorbent). It can involve both adsorption (the accumulation of molecules on the surface of the solid phase) and absorption (the phenomenon where molecules penetrate through the solid surface layer into the interior). In this study, the process by which GCBs acquire exogenous flavor compounds from a solution of flavor substances represents the sorption process of adsorbing sorbates using GCB as a sorbent [15]. Several factors can influence the sorption capacity of the sorbent, including time, temperature, and the microstructure of the sorbent. Moreover, this sorption process can be quantitatively described using kinetic models. Kinetic modeling is useful for the in-depth understanding of sorption characteristics, typically employing empirical equations such as pseudo-first-order and pseudo-second-order kinetics to characterize the sorption process [16,17]. However, there is currently no research on the sorption capacity of GCB as a sorbent for flavor substances.

The structure of GCB is important for both the introduction of flavor compounds and the roasting process; however, there is currently a paucity of research on this topic. Furthermore, as a thermo-sensitive material, the exposure time of GCB to high temperatures during the modification process must be considered. The objective of this study was to evaluate the effect of four physical pretreatments—hot air drying, freeze-drying, heating and puffing, and microwave and puffing—on the color change, porosity, and microstructure of GCB. The effect of the modified GCB structure on the sorption capacity as well as the extraction yield of caffeine and chlorogenic acid was also studied.

## 2. Materials and Methods

### 2.1. Green Coffee Beans (GCB)

GCB of Kenya (SL28 variety, provided by Saza Coffee Holdings Ltd., Hitachinaka, Japan) were used as samples. The GCB were pre-processed by wet processing and dried to the water content of 0.10 g/g (db).

### 2.2. Determination of Moisture Content of GCB at Different Soaking Times

An amount of 50 g of GCB was weighed and placed in 250 mL sterile glass media bottles, followed by adding 100 mL of distilled water. The beans were then soaked at room temperature for 0, 10, 20, 30, 60, 120, 180, 240, and 300 min. After soaking, the moisture content of the beans was measured by a moisture analyzer (MX–50, A&D, Tokyo, Japan). The linear form of the Peleg Equation (1) was applied to the water absorption data to model the hydration behavior.

(1) $$\frac{t}{M_{t}-M_{0}}=k_{1}+k_{2}t$$

where *M*_0_ is the initial moisture content (g/g), *M_t_* is the moisture content (g/g) at time *t*, *k*_1_ is the Peleg’s rate constant (min⋅g/g^−1^) and *k*_2_ is the Peleg’s capacity constant (g/g^−1^).

### 2.3. Modification of Pre–Soaked GCB

#### 2.3.1. Hot Air Drying (HD) and FREEZE–Drying (FD)

The first batch of the soaked GCB was placed in the dryer (WFO–500W, Tokyo Rikakikai Co., Ltd., Shanghai, China) and dried at 70 °C. The dryer is equipped with a forced-air circulation system, where the airflow not only circulates from top to bottom but also from the rear in parallel, ensuring a more uniform temperature distribution. The bed density was maintained at 0.25 g/cm^3^ throughout the drying process [18]. For the second batch of soaked GCB, the samples were pre-frozen at −20 °C for 12 h, then placed in the freeze dryer (DRC–3L, Tokyo Rikakikai Co., Ltd., Tokyo, Japan) and dried at a vacuum pressure of 40 Pa for 24 h. The drying chamber temperature was −40 °C, while the heating plate and cold trap were at 40 °C and −50 °C, respectively [19,20]. The hot air-dried GCB (HD–GCB) and freeze-dried GCB (FD–GCB) were stored in waterproof bags at 4 °C for further physicochemical analyzes. 

The basic principle of a freeze dryer is to remove water from frozen materials using the physical processes of freezing and sublimation [20]. Specifically, in the frozen state, water is frozen into ice crystals. Under low pressure, the ice crystals directly sublimate from a solid state to a gaseous state, thereby removing the water from the material and achieving drying. Due to the low temperatures used for freeze-drying, high-temperature evaporation-induced thermal damage to GCB can be avoided during the processing, while the quality of GCB can be maximally preserved.

#### 2.3.2. Short–Time Heating & Puffing (SHP) and Microwave & Puffing (MWP)

The pretreatments mentioned above mainly involved two approaches for altering the microstructure of GCB by removing moisture: evaporation and sublimation.

**Figure 1:**
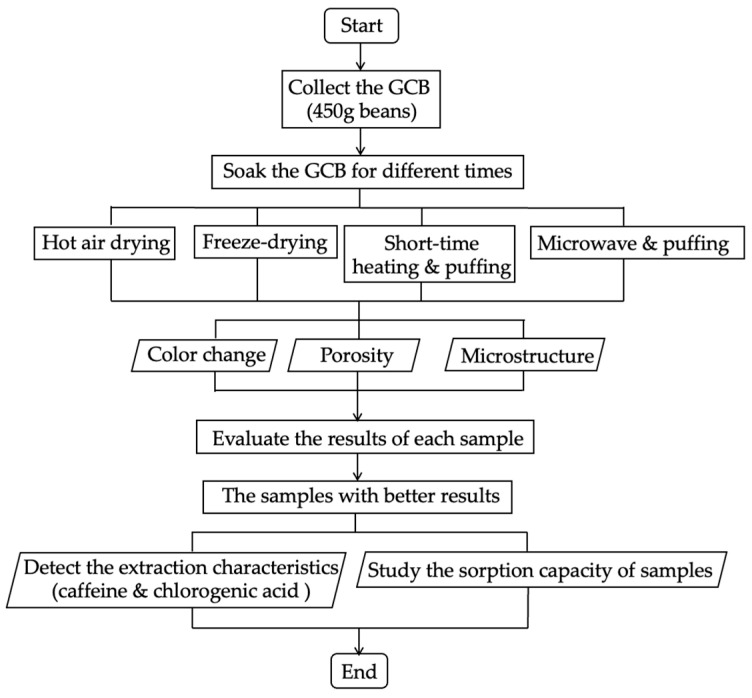
Pretreatments of green coffee beans.

This part demonstrated the puffing process based on two low-temperature methods, SHP and MWP. For SHP, the third batch of beans were placed in the chamber of the puffing machine (SL type, Tachibanakikou, Utsunomiya, Japan) at atmospheric pressure, and the autoclave was sealed. Heating was conducted using direct flame at the bottom for 1, 2, or 3 min while the chamber was uniformly rotated, and a selected pressure of 0.8 MPa was manually maintained during the heating period using an air compressor [21,22]. After 10 min, an abrupt pressure drop was carried out by rapid opening of the sealing lid. For MWP, the fourth batch of beans were placed into the microwave oven (500 W) (ER–VS23, Toshiba, Tokyo, Japan), and heated for 45, 60, and 75 s. Then, the heated beans were quickly transferred into the puffing machine chamber connected to the air compressor, and the pressure was raised to 0.8 MPa. After 10 min, the lid of the chamber was opened instantaneously. The short-time heated and puffed GCB (SHP–GCB) and microwave and puffed GCB (MWP–GCB) were stored in waterproof bags at 4 °C for analyses. The coffee beans subjected to the HD, FD, SHP, and MWP treatments were prepared in triplicate under the same conditions as parallel samples for repeated experiments. The brief flow of all the pretreatments is shown in Figure 1.

### 2.4. Color

A colorimeter (CR–200, Reston, Minolta, Tokyo, Japan) with a color difference (∆E*ab) within 0.15 was used to measure the color of GCB and treated GCB. The colorimeter was calibrated using a white calibration tile. Beans were evenly distributed on a petri dish. The values of L* (luminosity), a* (redness/greenness), and b* (yellowness/blueness) were determined using a standard illuminant A (incandescent lighting, correlated color temperature of 2856 K) and an observer angle of 2° [23,24]. Finally, the color differences were calculated using Formula (2).

(2) $$∆E^{*}=\sqrt{{\left(∆L^{*}\right)}^{2}+{\left(∆a^{*}\right)}^{2}+{\left(∆b^{*}\right)}^{2}}$$

where ΔE* represents the color difference between GCB and treated GCB.

### 2.5. Porosity

The porosity of beans refers to the porosity inside the beans, which is the ratio (varies from 0–100%) of the volume of void spaces or air pockets within the bean to the total volume of the bean. The true density (*ρ_g_*, g/cm^3^) and bulk density (*ρ_b_*, g/cm^3^) of coffee beans were measured using the pycnometer test method, and the porosity of the sample was calculated using Formula (3) [25].

(3) $$Porosity=\frac{\rho_{g}-\rho_{b}}{\rho_{b}}\times 100$$

### 2.6. Microstructures

The SEM employs an electron beam to scan the surface of the sample and obtain imaging results. If the moisture content of the sample is relatively high, the electrons in the beam may collide with water molecules that evaporate due to the vacuum environment of the SEM, resulting in loss of energy and direction, which can affect the accuracy and resolution of the imaging results [11]. Therefore, when observing the microstructure of the sample using SEM, the sample is usually required to be dry [12]. In this study, the final moisture content of dried beans, freeze-dried beans, and puffed beans (dried at 70 °C in an oven after puffing) were all unified to be 0.08 g/g (db).

The beans were cut and fixed to aluminum stubs using SEM mounting adhesive. The cross section of each sample was adjusted to the same horizontal position. All the samples, which were treated differently, were measured under vacuum at an accelerating voltage of 15 kV [26]. Finally, the samples were observed and photographed at a magnification of 250×. Finally, the pore size distribution of the modified coffee beans was analyzed using ImageJ software version 1.54.

### 2.7. Caffeine and Chlorogenic Acid Extraction Characteristics

For the extraction of caffeine and chlorogenic acid (CGA), beans treated by different pretreatments were ground and measured to 1.0 g and extracted with 50 mL of distilled water at 100 °C for 10 min while stirring at 1000 rpm. Centrifugation was performed at 4 °C, 14,000× *g* for 10 min. Standards of caffeine (1600 mg/L) and CGA (1600 mg/L) were prepared and diluted. Both samples and standards were filtered by a 0.45 μm membrane filter. The filtered samples and standards were stored at 4 °C for HPLC analysis. The samples were adsorbed onto the Shim-pack VP–ODS column (4.6 mm I.D. × 150 mm, 5 μm) and eluted by mobile phases A: 10 mM phosphate buffer (pH 2.6) and B: Acetonitrile at the flow rate of 1 mL/min. The elution program was as follows: 0–5 min 90% A, 5–15 min 90–30% A, 15–18 min 30% A, 18–18.01 min 30–90% A, 18.01–24 min 90% A. Finally, a spectrophotometer (V-630, Jasco, Tokyo, Japan) were used to determine the presence of caffeine and CGA in the samples at wavelengths of 270 nm and 320 nm, respectively. 

### 2.8. Sorption Studies

Citric acid solution (400 mg/L) was prepared by dissolving citric acid in distilled water. Sorption studies were conducted in triplicate to ensure reliability, following a consistent batch sorption procedure. Specifically, a known amount of modified GCB (1.0 g) was added to a 50.0 mL polyethylene tube, followed by the addition of 30.0 mL of citric acid solution. The effect of sorption time on the amount of sorption was examined at time intervals of 0, 30, 60, 90, 120, 150, 180, 210, 270, and 300 min. An individual vial was utilized for each duration of interaction time to ensure that changes in sample amount and solution volume caused by sampling would not affect the subsequent sorption process. Experiments were performed at room temperature, and all vials were shaken to equilibrate the standard citric acid solution and modified GCB during the sorption experiments [15,17]. The concentration of citric acid in the remaining solution within the vials was measured using citric acid kits (J.K. International, Tokyo, Japan) when the corresponding interaction time was up.

The sorption amount and time were fitted to pseudo-first-order equation:

(4) $$q_{t}=q_{e}\left(1-e^{{-k}_{1}t}\right)$$

The sorption amount and time were fitted to pseudo-second-order equation:

(5) $$q_{t}=\frac{{q_{e}}^{2}k_{2}t}{1+q_{e}k_{2}t}$$

where *q_t_* (mg/g) is the citric acid sorption amount at time *t* (min); *q_e_* (mg/g) represents the citric acid sorption amount at the equilibrium point (min); *k*_1_ (min^−1^) and *k*_2_ (g/mg min) are the rate constants of pseudo-first-order sorption and pseudo-second-order sorption.

### 2.9. Statistical Analysis

All experiments were carried out in triplicate, and data are reported as M ± SD deviation. Analysis of variance (ANOVA) was performed with a significance level set at *p* < 0.05, followed by post-hoc Tukey HSD using SPSS version 28.0.1.1 (IBM Corp., New York, NY, USA).

## 3. Results and Discussion

### 3.1. Moisture Content of Soaked GCB

**Figure 2:**
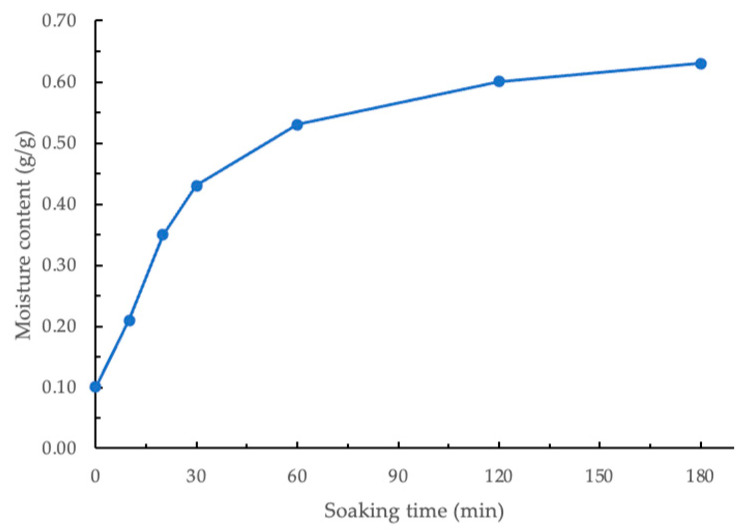
Moisture content of soaked coffee beans.

The moisture content of GCB soaked for different times is shown in Figure 2. The initial moisture content of the GCB processed by wet method and dried was 0.10 g/g (db). Statistical analysis showed a significant difference (*p* < 0.05) in the moisture content of beans soaked for different durations. As shown in Figure 2, the moisture content of beans increased rapidly during the first 30 min and then became relatively stable. Therefore, for subsequent modification experiments, beans soaked for 30, 60, 120, and 180 min were selected. Within the time range of 30–180 min, the derived constants *k*_1_ and *k*_2_ using the linearized Peleg regression model were 40.57 min⋅g/g^−1^ and 1.66 g/g^−1^, respectively. When GCBs are soaked in water, the microporous structure within the beans absorbs water through capillary action, allowing moisture to penetrate the beans quickly. This capillary action is particularly pronounced in the initial stage due to the moisture content gradient between the interior and exterior of the beans, driving the migration of water and rapidly increasing moisture content [27]. Additionally, the hydrophilic components in GCB, such as polysaccharides, proteins, and cellulose, bind to water, and their hydration further enhances water absorption by the beans [28].

### 3.2. Color Changes of Modified GCB

The results presented in Table 1 demonstrate the color changes in GCB subjected to different treatments and soaking times.

It was observed that the SHP treatment induced the largest color change, regardless of the moisture contents of GCB, especially when heated for 3 min. The color change, caused by MW_75_P treatment was also larger than that caused by MW_45_P and MW_60_P treatments. This suggests that heating time indeed has a significant impact on GCB color. MW_45_P and MW_60_P treatment also induced relatively large color changes but the effect of microwave for 45 s and 60 s on the color of GCB was significantly lower than that of short-time heating. This is because microwave heating is performed through internal molecular vibration and frictional heating, whereas short-time heating is performed from the outside to the inside.

Additionally, there was no clear trend in the color changes of GCB with different moisture contents subjected to the same treatment method. However, when there was no soaking before SHP treatment, the color changes were much greater than when there was a soaking step. This suggests that appropriately increasing the moisture content of GCB can prevent or reduce changes in bean color during non-roasting heating processes (which are accompanied by chemical changes). Monica Anese and Deborah Bauer mentioned that during roasting, the color of coffee beans gradually changes from light to dark, with the L* value decreasing and the a* and b* values increasing as roasting progresses. In addition, the ΔE* values of Robusta beans were 9.47 and 9.78 when roasted at 160 °C for 5 min and 215 °C for 10 min, respectively [29,30]. This change is primarily driven by non-enzymatic browning reactions. The Maillard reaction, occurring between reducing sugars (such as glucose) and amino acids, is a key mechanism responsible for the browning of coffee beans during heat treatment. Additionally, caramelization also contributes to color development, especially at higher temperatures [1,30]. The color change observed in the SHP–GCB and MW_75_P–GCB, although lower than the color change in roasted beans, was significantly greater than the color change of treated GCBs in the HD, FD, MW_45_P, and MW_60_P treatments. Since the SHP process involves heating with an open flame outside the chamber of the puffing machine, even though the final goal is not roasting, the GCBs (especially non-soaked GCB treated with SH_3_P) were slightly roasted due to the high temperature during processing. This could lead to the loss of previously formed volatile compounds during the final roasting (which can be considered a secondary roasting), resulting in an aroma profile of the final coffee that deviates from the expected characteristics [31]. Additionally, the color changes in MW_45_P–GCB and MW_60_P–GCB are significantly less than those reported by Dong [32]. In conclusion, observations of color changes indicate that HD, FD, MW_45_P, and MW_60_P have relatively minor effects on GCB.

### 3.3. Porosity of Modified GCB

The porosity of a bean refers to the proportion of pore space occupied within the bean. A higher porosity of coffee beans indicates a lower density, which corresponds to a softer texture. In addition, beans with higher porosity usually have better water sorption and retention properties [33,34]. The porosity of the GCB, soaked for 10, 20 min before being treated with different pretreatments, was also measured. However, due to the low moisture content inside, there was no significant change in porosity. This is also the reason why GCB soaked for 10, 20 min was not chosen for modification.

**Figure 3:**
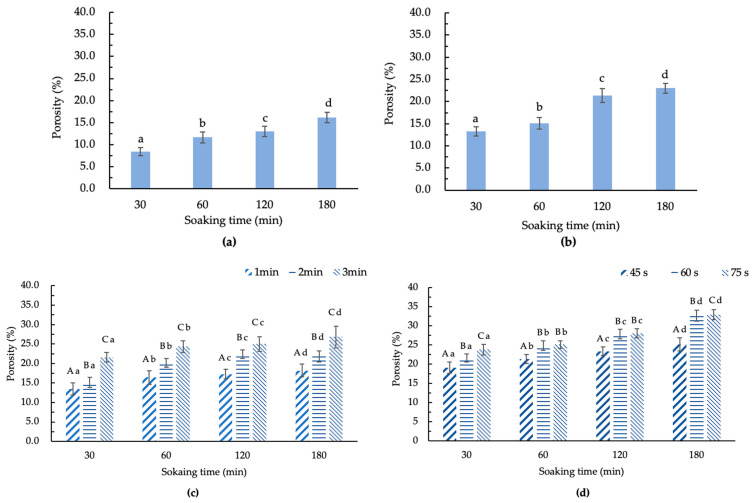
The porosity of modified beans by treatments. (**a**) HD–GCB; (**b**) FD–GCB; (**c**) SHP–GCB; (**d**) MWP–GCB. In (**a**,**b**), different letters in the same figure indicate significant differences (*p* < 0.05). In (**c**,**d**), different capital letters at the same soaking time and different heating times indicate significant differences (*p* < 0.05). Different lowercase letters at different soaking times and the same heating or microwave time indicate significant differences (*p* < 0.05). The sample size (*n*) was 3.

Figure 3 illustrates the porosity of GCB treated by different methods after soaking for different times. The untreated GCB had a porosity of 3.9%. As depicted in Figure 3a–d, it can be observed that under the same pretreatment, the porosity of treated GCB was related to the moisture content. When the moisture content increases, the porosity becomes larger. In addition, different pretreatments also have a significant impact on the porosity of GCB.

It can be observed that HD, FD, SHP, and MWP can all effectively increase the porosity of GCB. The porosity of GCB soaked for 180 min after HD, FD, SHP (1, 2, 3 min), and MWP (45, 60, 75 s) treatments were 16.1, 23.0, 18.2, 21.8, 26.8, 25.1, 32.6, and 32.9%, respectively. The porosity of GCB soaked for 30 min after HD, FD, SHP (1, 2, 3 min), and MWP (45, 60, 75 s) were 8.5, 13.3, 13.5, 15.2, 21.5, 19.1, 21.5, and 23.8%, respectively. This suggested that soaking, drying techniques, and puffing can significantly change the porosity of GCB. The reason for the small change in the porosity of HD–GCB may be that the drying temperature was only 70 °C, so the evaporation of moisture was relatively mild. Although the pressure in the chamber of the puffing machine for SHP and MWP was both 0.8 MPa, the porosity results were significantly different. This indicated that the violent shaking of molecules during the microwave heating process increases the effect of water evaporation, leading to changes in the microstructure of soaked GCB. The porosity of MW_45_P–GCB soaked for 30, 60, 120, and 180 min was 19.1, 21.3, 23.3, and 25.1%, respectively, which was significantly lower than that of MW_60_P–GCB soaked for the same times (*p* < 0.05). And the results of GCB treated by MW_60_P were better than those of GCB treated with an instantaneous controlled pressure drop process [11]. Therefore, MW_60_P can be mainly used to treat GCB before roasting, considering that this treatment does not cause significant changes in the color of the GCB and the porosity of GCB treated with MW_60_P treatment is higher.

### 3.4. The Microstructure of GCB and Modified GCB

**Figure 4:**
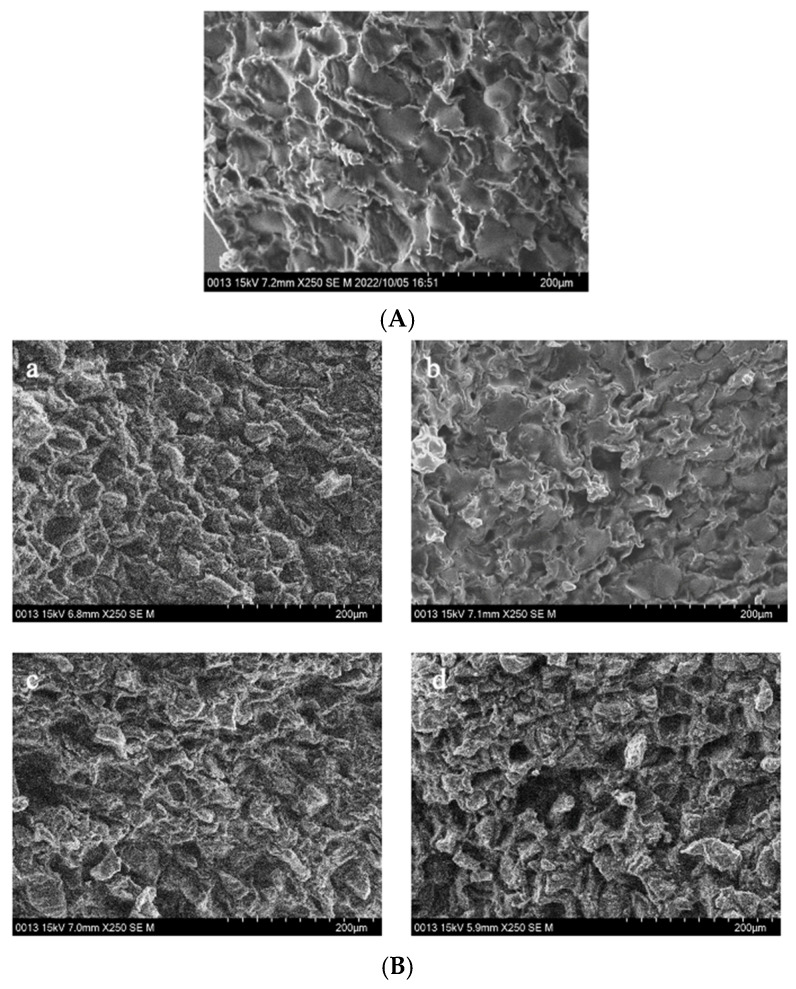

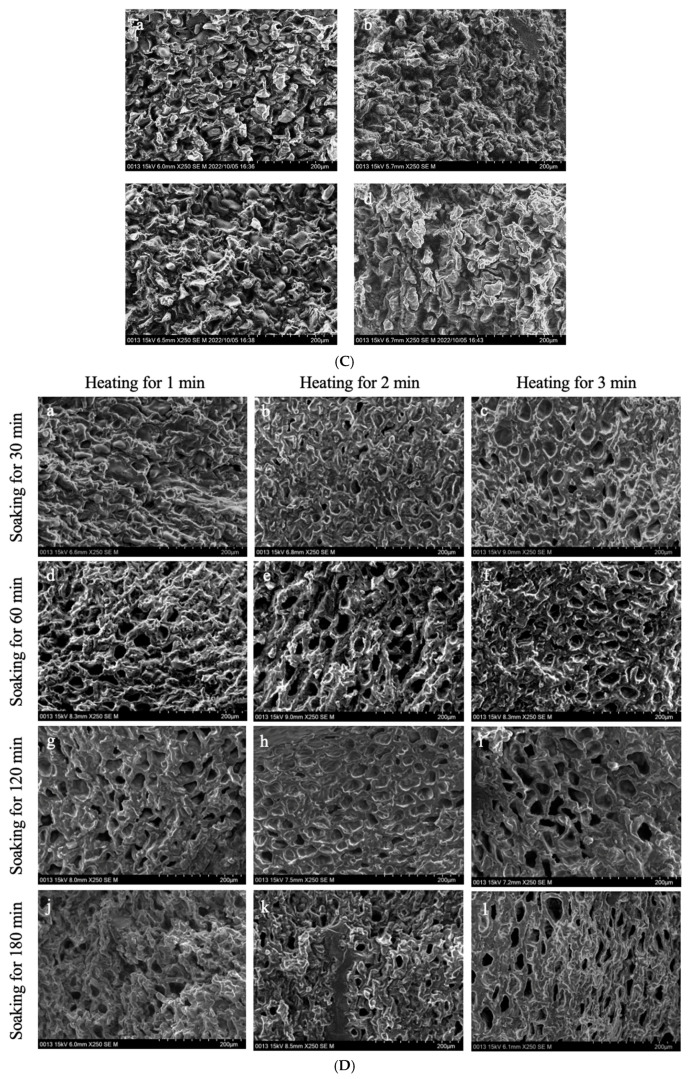

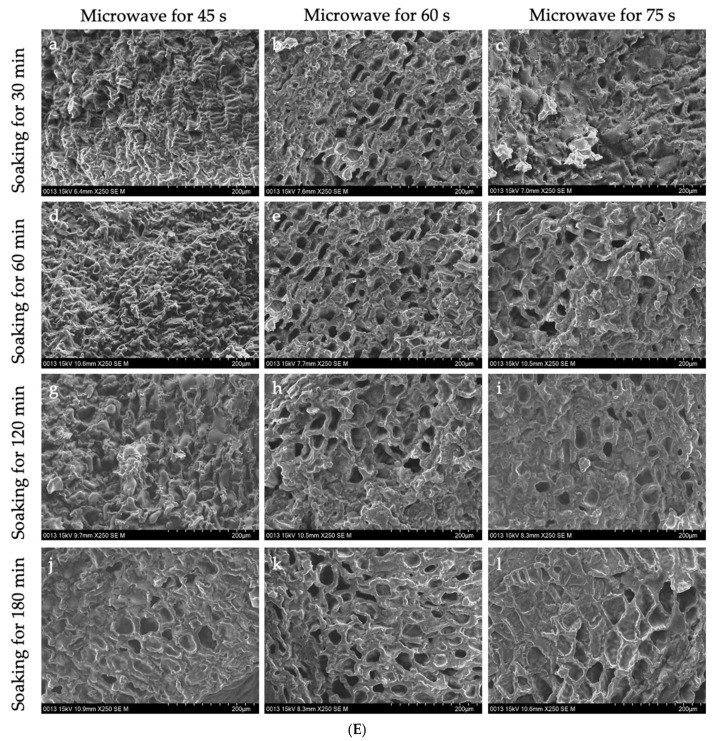
The interior of GCB and modified beans. (**A**) GCB; (**B**) HD–GCB; (**C**) FD–GCB; (**D**) SHP–GCB; (**E**) MWP–GCB. a, b, c, d showed in (**B**,**C**) mean GCB pre-soaked for 30, 60, 120, and 180 min then dried or freeze-dried. a–c, d–f, g–i, and j–l showed in (**D**,**E**) mean GCB pre-soaked for 30, 60, 120, and 180 min, respectively, followed by heating (for 1, 2, or 3 min) or microwave (for 45, 60, or 75 s).

Figure 4 shows the microstructure of GCB pre-soaked in water for different times, then subjected to the HD, FD, SHP, and MWP treatments.

**Figure A1:**
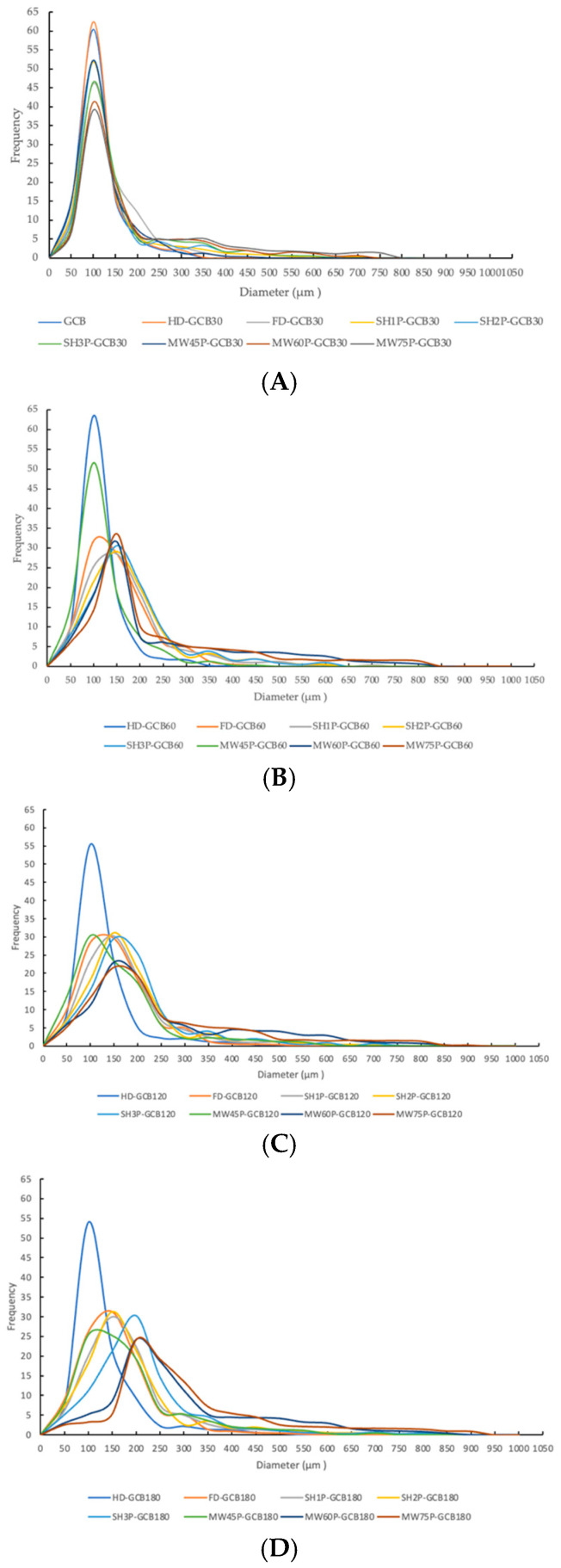
Pore size distribution of modified GCBs. (**A**) GCB and modified GCBs pre-soaked for 30 min; (**B**) modified GCBs pre-soaked for 60 min; (**C**) modified GCBs pre-soaked for 120 min; (**D**) modified GCBs pre-soaked for 180 min.

Figure 4A displays the cross-sectional microstructure of untreated GCB, which indicates a tight structure with few small pores. Pores with a diameter of 50–100 μm accounted for approximately 60.45% of the total (Figure A1). Figure 4(Ba–Bd) shows the microstructure of GCB pre-soaked for different times and then dried. The HD process had little effect on the structure of the GCB, but longer soaking time caused partial collapse of the internal structure. And most of the pores had diameters in 50–100 μm. Figure 4C shows the distorted and non-uniform internal structure of GCB after FD, which may be due to the formation of ice crystals that destroyed the internal tissue structure, thus affecting the uniformity of the microstructure. However, more pores with diameters in the 100–150 μm range were still observed (Figure A1). The kinetics of freezing influence the size and distribution of ice crystals formed within the beans. Rapid freezing tends to produce smaller ice crystals, resulting in finer pores and a more uniform pore size distribution upon thawing.

Figure 4D shows the microstructure of GCB after SHP. The structure of the beans changed significantly due to the heating and soaking time, particularly when heated for 3 min, which caused a high temperature in the puffing chamber and rapid evaporation of water inside the beans, resulting in the generation of dense and large pores. Among them, 66.1% of the pores in SH_3_P–GCB180 had diameters in the 100–250 μm (Figure A1). Figure 4E displays the microstructure of GCB after MWP. The experimental results demonstrate that the pore structure of MW_45_P–GCB was mostly distributed in the 50–150 μm (Figure A1), which was not as optimal as that observed in the SEM results of MW_60_P–GCB and MW_75_P–GCB. This is attributed to insufficient heating, which led to inadequate evaporation of moisture within the GCB, thereby preventing the formation of uniform pores. The porosity of GCB treated with MW_45_P at different soaking time was 19.1, 21.3, 23.3, and 25.1%, significantly lower than that of MW_60_P–GCB and MW_75_P–GCB. The SEM results for MWP–GCB confirmed the calculation results well. Dong subjected GCB to microwave treatment at different power levels (0.3 kW to 2.0 kW) for durations significantly longer than 1 min. The results showed that the treated GCB exhibited significant structural fragmentation [32]. However, structural fragmentation was not observed in the three types of MWP–GCB. Both MW_60_P–GCB and MW_75_P–GCB exhibited noticeable porous structures, with relatively large pore diameters, as pores with diameters in the 200–300 μm range accounted for 54.15% and 56.71% (Figure A1), respectively. Although MW_75_P–GCB exhibited a relatively good pore structure, its color change values were significantly higher than those of the other beans. GCB treated with MW_60_P displayed satisfactory microstructures with uniformly distributed and large pore diameters. In comparison, FD and MW_60_P can be considered an effective and convenient method for altering the microstructure of coffee beans.

### 3.5. CGA and Caffeine Extraction Characteristics

Due to the significantly lower porosity of MW_45_P–GCB compared to MW_60_P–GCB and MW_75_P–GCB, as well as the significantly greater color changes observed in SHP–GCB and MW_75_P–GCB compared to the other modified beans, the extraction characteristics of caffeine and CGA in these beans were not analyzed.

**Figure 5:**
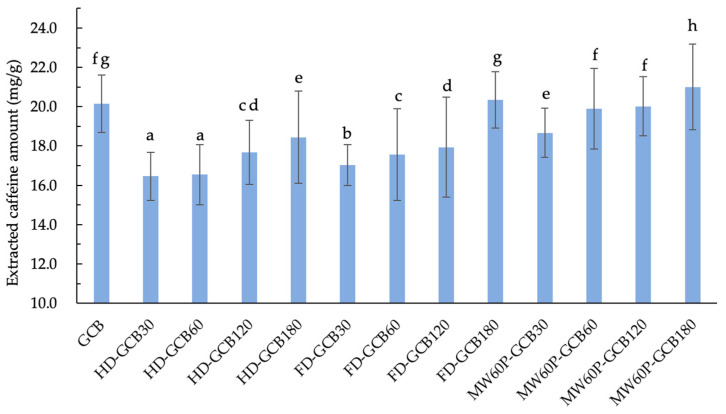
Content of extracted caffeine in GCB and modified beans. The number after the GCB means the pre-soaking time (30 min, 60 min, 120 min, and 180 min) before different treatments. Different letters in the same figure indicate significant differences (*p* < 0.05). Error bars represent standard deviations of *n* = 3 samples.

Figure 5 illustrates the extraction amount of caffeine from GCB and treated GCBs under the same extraction conditions. It can be observed that soaking and treatments generally result in a loss of total caffeine in GCB. However, the extracted caffeine contents of FD–GCB180, MW_60_P–GCB60, and MW_60_P–GCB120 were not significantly different from GCB (20.2 mg/g), while the caffeine content extracted from MW_60_P–GCB180 (20.9 mg/g) was significantly higher than that of GCB (*p* < 0.05). The current results are consistent with a previous study by Kim [10]. This is attributed to the fact that, despite the overall reduction in the total caffeine content in the coffee beans, the microstructure of the pretreated coffee beans has become more porous, with an increased number of pores distributed in the range of more than 200 μm. Consequently, the yield of caffeine extraction increases, resulting in a higher overall caffeine extraction yield.

**Figure 6:**
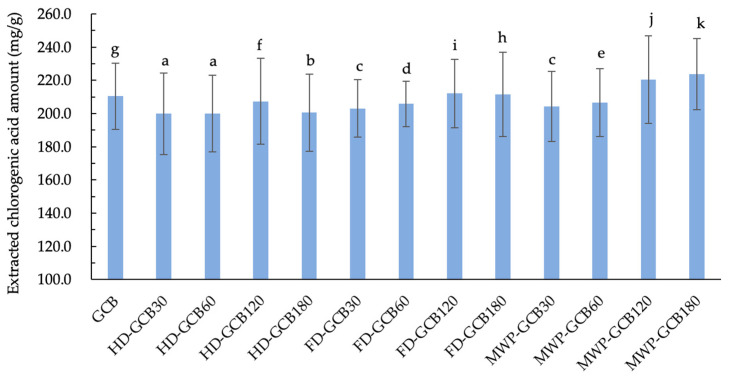
Content of extracted chlorogenic acid in GCB and modified beans. Different letters in the same figure indicate significant differences (*p* < 0.05). Error bars represent standard deviations of *n* = 3 samples.

The result shown in Figure 6 illustrates the extraction amount of CGA from GCB and treated GCBs. It is evident that soaking and treatments indeed result in a loss of CGA in GCB. The extracted CGA amount for HD–GCB, FD–GCB30, FD–GCB60, and MW_60_P–GCB30, and MW_60_P–GCB60 was significantly lower than that of GCB (*p* < 0.05). However, due to the longer soaking time and appropriate treatment, the microstructure of GCBs becomes more porous. Therefore, the extraction amount of CGA from some beans with a looser structure was greater than that of GCB. For example, the extraction amounts of CGA from MW_60_P–GCB120, and MW_60_P–GCB180 (with 59.83% and 83.11% of pores larger than 200 μm) were 220.4 and 223.8 mg/g, respectively, which were significantly higher than those in GCB (210.4 mg/g) (*p* < 0.05).

Due to soaking and treatments, coffee beans lost part of the caffeine and CGA, leading to an overall reduction in the total content of caffeine and CGA [35,36]. However, these treatments altered the microstructure of the coffee beans. Studies have shown that pre-soaking for 180 min then MW_60_P can significantly increase the porosity of the material and improve the pore size distribution, thereby enhancing the yield of the extract. Additionally, Liu and Bilge have mentioned in their research on pulse electric field (PEF) treatment of tea leaves and Arabica GCB that the disruption of plant cells can facilitate increased extraction yield. The increase in porosity of tea leaves and the formation of surface protuberances may improve the ability of the solvent to penetrate the cells and the yield of extract diffusion across cell membranes [14,37]. When the pore size distribution is appropriate, caffeine and CGA molecules can pass through the pores into the solvent more quickly, and this rapid diffusion significantly enhances extraction. Additionally, larger pores also allow the solvent to flow within the beans, facilitating the dissolution process.

### 3.6. Sorption Kinetics

**Figure 7:**
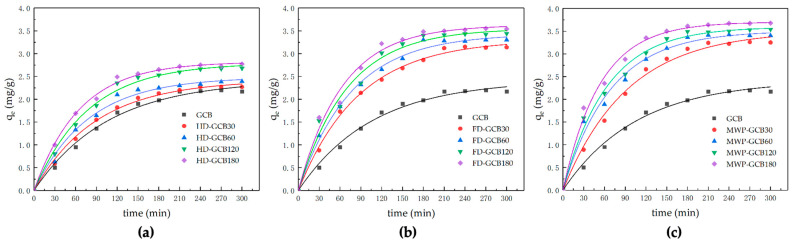
Kinetic modeling of the sorption process of modified GCB for citric acid. (**a**) HD–GCB; (**b**) FD–GCB; (**c**) MWP–GCB.

The kinetic models and corresponding parameters are shown in Table 2. The amount of citric acid adsorbed by HD–GCB, FD–GCB, and MW_60_P–GCB at time t versus the time is presented in Figure 7.

As the porosity of modified GCB increased from 3.90% to 32.64%, the experimentally measured sorption capacity of citric acid, *q_e,exp_*, increased from 2.21 to 3.68 mg/g. The sorption of citric acid onto GCB and modified GCB followed the pseudo-first-order kinetic model, as the predicted equilibrium sorption capacity calculated by the pseudo-first-order model showed better agreement with the experimental sorption capacity compared to the pseudo-second-order model. This is further supported by the higher *R*^2^ (0.98874–0.99657) of the pseudo-first-order model. Mi–Hwa Baek used degreased coffee beans with a similar porous structure as sorbents to adsorb malachite green and described the sorption process using both first-order and second-order models. They reported a similar trend in the change of sorption amount over time [15]. The equilibrium sorption capacity, *q_e_*, increased from 2.41 to 3.70 mg/g, and the value of *k*_1_ increased from 0.00943 to 0.01862 min^−1^ when the microstructure of the modified GCB became more porous. Previous literature reported that the pore structure of the sorbent can influence the sorption rate [38,39]. Although the first-order and second-order pseudo kinetic models are regarded as empirical equations and do not reflect the actual chemical and physical phenomena occurring, they remain valuable as simple equations for predicting the kinetics of sorption systems.

Citric acid is generally considered to show citrus and acidic flavor and can assist with flavor modification as well as increasing cupping scores by spontaneous fermentation [40]. Na and Martinez introduced yeast and lactic acid bacteria during the fermentation of GCB, fermenting the GCB for 48 h. Although the citric acid content in the fermented GCB increased, the pattern of its variation remained unpredictable [41,42]. This study utilized modified GCB as sorbents to adsorb flavor compounds, achieving not only an enhancement in sorption capacity but also the visualization and predictability of the adsorbed flavor compound quantities.

## 4. Conclusions

In conclusion, this study demonstrated that pretreatment methods, specifically MW_60_P, significantly enhanced the porosity and pore size distribution of GCB, leading to improved extraction yields of caffeine and chlorogenic acid. The experimental results indicated a clear relationship between the microstructural modifications and the increased sorption capacity for citric acid, providing insights for effectively introducing exogenous flavor compounds into GCB before roasting.

The generalizability of the results may be limited by specific experimental conditions, such as the selection of coffee bean varieties. Future research should focus on exploring a broader range of coffee bean varieties. Furthermore, the established relationship between changes in microstructure and the sorption capacity of flavor compounds provides a framework for future research aimed at optimizing coffee flavors through non-fermentation methods. Researchers can investigate the sorption characteristics of modified coffee beans concerning different flavor components to achieve targeted alterations of desired flavor attributes, ultimately enhancing the consumer experience.

## Figures and Tables

**Table 1:** The color change (ΔE*) of modified green coffee beans (M ± SD).

Treatment	Soaking Time (min)				
	0	30	60	120	180
HD	1.87 ± 0.12 ^Cb^	1.43 ± 0.09 ^Aa^	1.57 ± 0.05 ^ABab^	1.72 ± 0.14 ^BCab^	1.79 ± 0.16 ^BCb^
FD	0.85 ± 0.18 ^Aa^	1.24 ± 0.22 ^Ba^	1.31 ± 0.28 ^Ba^	1.33 ± 0.11 ^Ba^	1.26 ± 0.18 ^Ba^
SH_1_P	4.47 ± 0.67 ^Be^	3.38 ± 0.34 ^Ac^	3.61 ± 0.45 ^Ad^	3.89 ± 0.33 ^ABb^	3.62 ± 0.25 ^Ae^
SH_2_P	6.53 ± 0.49 ^Cf^	4.97 ± 0.33 ^Ad^	5.14 ± 0.34 ^ABe^	5.78 ± 0.12 ^Bc^	5.69 ± 0.32 ^Bf^
SH_3_P	8.17 ± 0.77 ^Ag^	6.38 ± 0.34 ^Be^	6.01 ± 0.25 ^Bf^	5.89 ± 0.23 ^Bc^	5.78 ± 0.25 ^Bf^
MW_45_P	3.02± 0.43 ^Cc^	2.14 ± 0.19 ^Bb^	1.88 ± 0.24 ^Abc^	2.29 ± 0.21 ^Aab^	2.35 ± 0.43 ^Ac^
MW_60_P	3.17 ± 0.55 ^Cc^	2.23 ± 0.13 ^ABb^	2.01 ± 0.11 ^Ac^	2.47 ± 0.08 ^ABab^	2.59 ± 0.12 ^Bc^
MW_75_P	4.17 ± 0.33 ^Cd^	3.54 ± 0.32 ^Bc^	3.69 ± 0.42 ^Bd^	3.05 ± 0.25 ^Aab^	3.14 ± 0.29 ^Ad^

SH_1_: short-time heating for 1 min; SH_2_: short-time heating for 2 min; SH_3_: short-time heating for 3 min; MW_45_P: microwave for 45 s; MW_60_P: microwave for 60 s; MW_75_P: microwave for 75 s. ^A–C^ In the same row, means with different capital letters are significantly different (*p* < 0.05). ^a–g^ In the same column, means with different lowercase letters are significantly different (*p* < 0.05).

**Table 2:** Pseudo-first-order and pseudo-second-order fitting parameters of GCB and modified GCB for citric acid.

Sample	Pre–Soaking Time	*q_e,exp_*	Pseudo–First–Order			Pseudo–Second–Order		
			*k*_1_	*q_e_*	*R* ^2^	*k*_2_	*q_e_*	*R* ^2^
GCB	/	2.21	0.00943	2.41	0.99173	0.00238	3.31	0.98414
HD–GCB	30	2.31	0.01116	2.42	0.99589	0.00317	3.19	0.98775
	60	2.39	0.01279	2.48	0.99007	0.00383	3.19	0.97874
	120	2.68	0.01285	2.80	0.99354	0.00348	3.57	0.98329
	180	2.77	0.01523	2.82	0.99633	0.00471	3.47	0.98776
FD–GCB	30	3.14	0.01151	3.31	0.99657	0.00251	4.29	0.99272
	60	3.31	0.01307	3.43	0.99431	0.00307	4.32	0.99039
	120	3.45	0.01428	3.55	0.99012	0.00353	4.37	0.97946
	180	3.54	0.01571	3.62	0.98957	0.00411	4.40	0.98128
MWP–GCB	30	3.25	0.01077	3.51	0.99302	0.00206	4.66	0.98424
	60	3.44	0.01455	3.52	0.98874	0.00371	4.30	0.98731
	120	3.54	0.01584	3.59	0.99133	0.00416	4.34	0.99051
	180	3.68	0.01862	3.70	0.99311	0.00527	4.36	0.99193

## Data Availability

The original contributions presented in the study are included in the article, further inquiries can be directed to the corresponding author.
